# Chronic bilateral hearing loss in an immunocompetent patient: An atypical course of tuberculous meningitis

**Published:** 2014-10

**Authors:** Asli G. Akyol, Bijen Nazliel, Yusuf Oner, Ozlem Erdem

**Affiliations:** *From the Departments of Neurology (Akyol, Nazliel), Radiology (Oner), and Pathology (Erdem), Faculty of Medicine, Gazi University, Besevler, Ankara, Turkey*

## Abstract

Congenital, infectious, toxic, and demyelinating disorders are common etiological causes of deafness. Tuberculous meningitis, as one of the infectious causes, should be considered in the differential diagnosis since tuberculosis represents an endemic public health problem in developing countries. Multiple cranial nerve palsies can be expected due to basal meningitis; however, presentation with bilateral hearing loss is quite rare. Early diagnosis and treatment are crucial to prevent mortality and residual neurologic deficits. The focus of this discussion is a 42-year-old female presenting with bilateral hearing loss and nonspecific complaints who was finally diagnosed with chronic tuberculous meningitis. We also demonstrate the characteristic radiological and histopathological findings.

Congenital, infectious, toxic, vascular, tumoral, and demyelinating disorders are common etiologic factors that can result in deafness. Acoustic trauma, endolymphatic hydrops, and senility are relatively rare causes that should also be taken into account. Bilateral hearing loss is an unusual clinical presentation, resulting from any one of these conditions. Among the infectious causes, tuberculosis still represents an endemic public health problem in developing countries and tuberculous meningitis, which is the most common form of CNS involvement, accounts for 1-2% of these cases.^1^ Sufferers generally describe constitutional symptoms including fever and night sweats or various complaints arising from basal meningitis like headache, nausea, and/or vomiting; however, presentation with bilateral hearing loss is quite rare. The objective of this case presentation is to include tuberculous meningitis in the differential diagnosis of progressive bilateral hearing loss, and emphasize the importance of early diagnosis and treatment to prevent morbidity and mortality.

## Case Report

**Figure 1:**
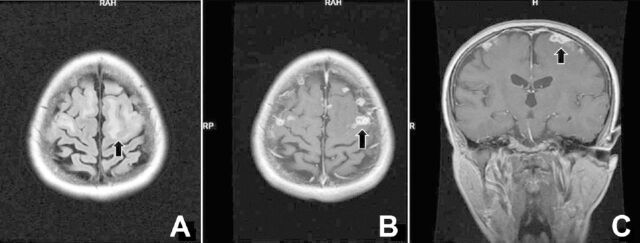
Patient MRI showing **A**) axial flair, **B**) postcontrast T1 weighted axial, and **C**) coronal images showing bilateral multiple enhancing tuberculomas at parietal lobes with accompanying edema.

**Figure 2:**
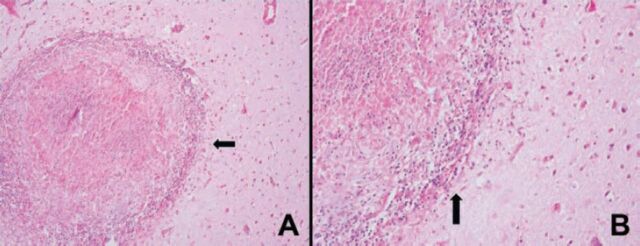
Necrotizing granulomatous inflammation in glial tissue, and caseous necrosis in the center of the granuloma, **A**) Hematoxylin eosin staining X200, and **B**) Hematoxylin eosin staining X400.

**Figure 3:**
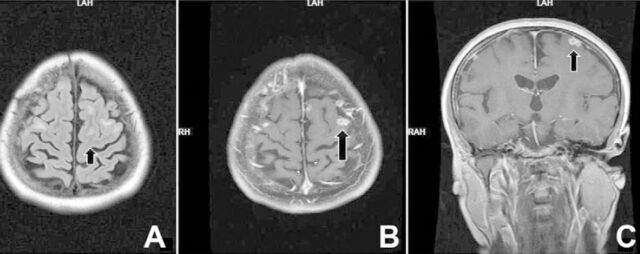
Patient MRI showing **A**) Axial Flair, **B**) postcontrast T1 weighted axial, and **C**) coronal images acquired at the same plane as figure 1 after 6 months of treatment. There is a decrease in size and number of tuberculomas, together with decreasing edema.

A 42-year-old female complained of progressively worsening headache, vertigo, tinnitus and bilateral hearing loss for 3 years. The headache was occasionally complicated with nausea and vomiting. She was first examined in the neurosurgery clinic and then referred to neurology as a cranial MRI performed in another center revealed the presence of T2 hyper-intensities suggestive of demyelinating lesions. She had been treated for infertility over the last 2 years and had delivered a healthy child 6 months ago. The familial and personal medical history was otherwise, unremarkable. Her neurologic examination was normal except for bilateral hearing loss. Her audiogram revealed bilateral sensorineural hearing loss while re-imaging of the brain, 2 months after the initial MRI, detected multiple cystic lesions on the right and left frontal convexity accompanied by pathological meningeal enhancement (**Figure 1**). Bilateral symmetric vasogenic edema in the frontoparietal and temporal regions was also present. An ECG and a chest x-ray showed normal findings. Results of complete blood count, serum biochemistry, erythrocyte sedimentation rate (5 mm/h) and C reactive protein (8 mg/dL) were within normal limits, and markers of vasculitis (except for a slightly positive nucleolar pattern of fluorescent antinuclear antibody) were also normal. A comprehensive blood survey including viral hepatitis markers; toxoplasma, rubella, cytomegalovirus, and herpes (TORCH) IgM, venereal disease research laboratory and rapid plasma reagin test, treponema pallidum hemagglutination test, human immunodeficiency virus (HIV) serology, *Echinococcus granulosus* IgG, and the indirect hemagglutination test were negative. The Rose Bengal test for brucella was positive, but the Wright and the 2-mercaptoethanol-agglutination test revealed negative results, as did the group agglutination (Vidal) test. Tuberculin skin test, blood, and urine cultures were also negative. A lumbar puncture was performed and the CSF was observed to be clear and colorless, while further analyses showed normal cell count (range: 0-10 leukocytes) with normal protein (35.8 mg/dL) and glucose levels (63 mg/dL; simultaneous serum glucose levels were 104 mg/dL). Microbiological staining revealed no gram-positive or -negative bacteria or acid-fast bacilli. Detailed investigation of CSF, including mycobacterium and Brucella polymerase chain reaction (PCR), and Brucella tube agglutination were all negative; however, Rose Bengal was uncertainly positive. Neither *Mycobacterium tuberculosis* nor other bacteria were isolated from the CSF culture. A minor salivary gland biopsy was performed due to her non-specific ocular complaints of burning and itching, and Sjogren’s syndrome was ruled out through the normal findings of histopathological assessment. An abdominal ultrasonography was also normal. Since current clinical and complementary data were not enough for a definite diagnosis, a cranial biopsy was performed from the right frontal subdural lesion and following the procedure, she was started on Rifampicin (600 mg/day), doxycycline (200 mg/day), and ceftriaxone (4000 mg/day) empirically with the early diagnosis of neurobrucellosis. The histopathological examination demonstrated necrotizing granulomatous inflammation with a holding caseous center, compatible with tuberculosis (**Figure 2**). The Ziehl-Neelsen stain and Kinyoun stain for acid-fast microorganisms and light green Periodic Acid-Schiff’s stain for fungus were all negative. Doxycycline was stopped, and isoniazide (300 mg/day), ethambutol (2000 mg/day), and pyrazinamide (2000 mg/day) were added to her treatment, with a final diagnosis of CNS tuberculosis. She was discharged from the clinic and recommended a cochlear implantation for bilateral sensorineural hearing loss after eradication of the infection. Antituberculosis treatment was continued for 12 months, and she was reevaluated clinically and radiologically, every 6 months. Her headache and accompaniments improved gradually, as did the lesions on the cranial MRI (**Figure 3**). Unfortunately, no improvement in the hearing loss was obtained.

## Discussion

The etiologic evaluation performed in this case, on an individual who presented with bilateral hearing loss; excluded acoustic trauma, diuretics, acetylsalicylic acid, side effects of streptomycin, as well as other toxic exposure using the medical history, and a demyelinating, vascular, or tumoral etiology was excluded with the aid of cranial MRI. An audiological evaluation, and the chronic, progressive course of complaints, which were free from remission periods, enabled Meniere’s disease and primary labyrinth disorder to be discounted as the responsible factor. Central nervous system infections are well-known causes of sensorineural hearing loss. The serological evaluation excluded syphilis, echinococcus, brucella, and a viral etiology, such as TORCH.

Tuberculosis is still an important health problem in developing countries, and the incidence in industrialized countries is also increasing because of the expanding HIV epidemic. The most prevalent agent responsible for clinical manifestations is *Myobacterium tuberculosis*. However, *Mycobacterium bovis* or atypical mycobacteria may occasionally be encountered. Central nervous system tuberculosis comprises 1-2% of all tuberculosis cases, and 8% of extrapulmonary forms among non-immunocompromised patients. The CNS involvement can occur in 3 categories; the most common form is septic meningitis, followed by intracranial tuberculoma, and spinal arachnoiditis.^1^ Sensorineural hearing loss in neurotuberculosis can be caused by meningitis leading to a spread of infection to the inner ear through the modiolus and cochlear aqueduct or tuberculous otitis media.^2^,^3^ Malaise, weight loss, low-grade fever, headache, and vomiting are known to be the most common symptoms.^4^ Multiple cranial nerve palsies, especially the involvement of the sixth cranial nerve, is expected because of the presence of basal meningitis; however, presentation with bilateral hearing loss is quite rare. Mild anemia and leukocytosis can be present, but complete blood count, and erythrocyte sedimentation rate are usually normal. Although obtaining a positive tuberculin skin test is non-specific, it can support the diagnosis; however, the reaction is commonly absent in all forms of active tuberculosis.^1^ The characteristic CSF features include straw colored macroscopic appearance, leukocytosis with a predominant lymphocytosis, low CSF:blood glucose ratio, and increased protein level;^5^ however, 25% of patients have less than 100 mg/dL proteins, and 15% have a cell count less than 100/mm^3^.^1^ The specific diagnosis depends on the isolation of *Mycobacterium tuberculosis* through CSF culture or microbiological staining. Culture confirmation requires a long incubation period and these conventional tests are often unhelpful in reaching the diagnosis, since tuberculous meningitis is a paucibacillary form of tuberculosis.^6^ The incidence of significant chest x-ray findings does not exceed 30%, even in the large cohorts of patients of neurotuberculosis without any pulmonary symptoms.^7^ Besides, meningitis and tuberculoma(s),^8^ brain CT and MRI demonstrate the manifestations of atypical complications including hydrocephalus, cerebritis or infarctions.^5^

Our patient had complaints of progressive hearing loss, tinnitus, and non-specific symptoms consisting of headache, nausea, and vomiting. The absence of fever can be attributed to the prolonged host defense mechanism during the chronic course of the disease. The chest x-ray was not typical, and the CSF demonstrated no abnormality. Although multiple cystic lesions accompanied by dural enhancement on the MRI suggested chronic meningitis; the atypical long course of her symptoms, absence of fever, and unremarkable results of CSF investigation complicated the diagnosis. A biopsy was performed and histopathological examination of the specimen from the subdural lesion confirmed the diagnosis of tuberculosis.

Since the sensitivity of the method is known to be as low as 56%,^6^ negative PCR results do not exclude the diagnosis of tuberculosis, even in the absence of clinical findings (for example, positive smear) sufficient enough to start empirical treatment. Because the reported fatality rates are as high as 28% (65% when serology for HIV is positive) and delays in diagnosis and treatment are the major contributing factors of high mortality; the clinician should immediately consider the diagnosis and start empirical treatment.^9^ The use of steroids as an adjunctive therapy in tuberculous meningitis improves the survival, probably via attenuation of the inflammatory response.^9^ The recommended duration of treatment in tuberculous meningitis is 9-12 months with the combination of 4 drugs (rifampicin, isoniazide, pyrazinamide, and streptomycin, or ethambutol) for the intensive phase, which is followed by a continuation phase with 2 drugs (rifampicin, isoniazide).^10^

Our patient was treated with isoniazide, pyrazinamide, ethambutol, rifampicin, and pyridoxine. Due to vestibulocochlear toxicity, streptomycin was not administered. A follow up MRI, which was performed 6 months after discharge, indicated the regression of tuberculomas; however, the hearing loss persisted.

Tuberculous meningitis has been discussed as an etiologic cause of sensorineural hearing loss rather than a fatal clinical condition, after the improvement of antimicrobial therapy in the late 1950’s. Since then, many reviews and case series, particularly in the pediatric population referred tuberculous meningitis and related hearing loss. However, the vast majority of these reports mentioned deafness as part of multiple cranial nerve palsies within the severe course of already diagnosed meningitis, a neurologic sequela in survivors after the treatment or an iatrogenic condition due to the acoustic toxicity of streptomycin. Until 2001, when Kotnis et al^3^ reported a 60 year old man suffering from unilateral sensorineural hearing loss who was finally diagnosed with tuberculous meningitis, deafness was not mentioned as the initial symptom. A comprehensive literature search did not reveal a case with progressive bilateral hearing loss as the presenting symptom. Contrary to common belief, tuberculosis may have a long progressive course with nonspecific symptoms and signs, especially in endemic regions. Early diagnosis and treatment are essential to prevent mortality and residual neurologic deficits.

In conclusion, tuberculosis should always be taken into account in the differential diagnosis of individuals, residing in undeveloped or developing countries and presenting with hearing loss.
